# Gender differences in footwear characteristics between half and full marathons in China: a cross-sectional survey

**DOI:** 10.1038/s41598-023-39718-x

**Published:** 2023-08-10

**Authors:** Yuyu Xia, Siqin Shen, Sheng-Wei Jia, Jin Teng, Yaodong Gu, Gusztáv Fekete, Tamás Korim, Haotian Zhao, Qiang Wei, Fan Yang

**Affiliations:** 1 School of Social Sciences, Tsinghua University, Beijing, China; 2 Faculty of Sports Science, Ningbo University, Ningbo, China; 3 Faculty of Engineering, University of Pannonia, Veszprém, Hungary; 4 Savaria Institute of Technology, Eötvös Loránd University, Szombathely, Hungary; 5 Li Ning Sports Science Research Center, Li Ning (China) Sports Goods Company Limited, Beijing, China; 6 Department of Sports Biomechanics, Beijing Sport University, Beijing, China; 7 Department of Materials Engineering, Faculty of Engineering, University of Pannonia, Veszprém, Hungary; 8 Department of Physical Education, Jiangnan University, Wuxi, 214122 China; 9 Department of Physical Education, Tangshan Normal University, Tangshan, China; 10 Department of Physical Education and Research, China University of Mining and Technology-Beijing, Beijing, 100083 China

**Keywords:** Orthopaedics, Rehabilitation, Bone quality and biomechanics, Muscle, Quality of life, Lifestyle modification

## Abstract

There are concerns about the risk of injuries caused by marathons in China. Since male and female runners have different injury risks, gender differences in running shoe functionality should be further complemented. A supervised questionnaire survey of 626 marathon runners was collected. The questionnaire was categorized into four sections: (1) participant profile, (2) importance of shoe properties, (3) functional evaluation of shoe properties and (4) importance ranking of shoe properties. The Mann–Whitney *U* test, Fisher’s exact test of cross tabulation and Chi-square test, and two-way ANOVA were used to analyze the results of this survey. The significance level was set at *P* < 0.05. The full marathon participants were older than the half marathon participants. There was no gender difference in the importance of shoe features to elite runners. In addition, women are more concerned about upper elasticity and have higher requirements for running shoes than men. Women were more focused on injury prevention, while men were more focused on running performance. Heel cushioning was identified by all participants as the most important running shoe feature. There were no gender differences between elite players’ demand for running shoes, but significant gender differences were found between genders at other running levels.

## Introduction

In recent years, marathon running has gained tremendous popularity in China. The number of national marathon and road running events surged from 51 in 2014 to a staggering 1828 in 2019, representing an increase of over 30 times in just five years. Furthermore, the total number of participants reached an impressive 7.12 million, showing a significant 22.22% increase compared to the previous year^1^. This surge in participation reflects the fact that marathons are no longer limited to talented runners but have become inclusive, attracting individuals of all ages and skill levels^2^. However, alongside the rise in popularity and participation, the occurrence of long-distance running-related injuries has also increased^3^. Consequently, scholars have directed their attention towards studying the biomechanics, performance, and sports equipment related to running^4–6^. Among the various factors that impact running, the choice of running shoes has emerged as a critical consideration for runners^7^. I In marathons, running shoes serve the primary purposes of protecting runners' feet from friction and cushioning the impact force generated during ground contact. This impact force can reach levels ranging from 2 to 5 times the body weight, potentially leading to running-related injuries^8–10^. Research has demonstrated that altering footwear properties can influence the movement characteristics of runners, thereby affecting both their sports performance and the risk of injuries^11–13^, For instance, tuning the forefoot longitudinal bending stiffness of running shoes can reduce energy loss in lower limb joints and improve overall running performance^11^; Similarly, increasing midsole thickness has been found to enhance the moment arm of the lower extremity, optimizing the running mechanism and improving running economy^12,13^.

In addition to the increasing popularity of marathon running, there has been a notable rise in the number of female participants. In 2020, over 50 million Americans participated in running or jogging, with only 9% of the participants being male (Rizzo, N. Statistics. 120 + Running Statistics 2021/2022. Available online: https://runrepeat.com/research-marathon-performance-across-nations (accessed on 7 March 2020).). Studies suggest that long-distance running strategies should be tailored based on gender, age, and the specific event a runner is training for^14^. Males and females exhibit differences in anatomical characteristics in long-distance running. Female runners tend to demonstrate a greater range of movement in their hip and knee joints compared to male runners, which results in lower joint stability for females compared to males^15^. These findings highlight the distinct needs of males and females when it comes to sports equipment. However, current footwear developers primarily produce female running shoes based on scaled-down versions of male lasts, which is an unreasonable approach for female runners. It is evident that shoe construction should consider the differences in foot shape and running characteristics between males and females, as well as the specific demands of runners^16–18^. It is evident that such an approach is unreasonable for female runners. Shoe construction should take into account the differences in foot shape and running characteristics between males and females, as well as the specific demands of the runners^8,16,19^. In comparison to the large number of participants in distance running, only a select few individuals, such as footwear designers and manufacturers, have the expertise to design and determine the construction of running shoes^20^. While there is an abundance of studies and theories on the biomechanical aspects of Chinese long-distance running, such as kinetics and lower limb kinematics^15,16,21,22^. While previous studies have investigated the characteristics of sports shoes for various activities such as gym workouts, football, basketball, tennis, and badminton using questionnaires^23–27^, limited information is available regarding the specific requirements of running shoes for marathon runners. Therefore, the self-perception of marathon runners when wearing running shoes remains an important aspect that requires further investigation and analysis. Understanding how marathon runners perceive the characteristics of their running shoes is crucial for designing footwear that meets their specific needs and enhances their overall running experience and performance.

Therefore, the purpose of this cross-sectional study is to examine gender differences in the perception of running shoe requirements among participants of different performance levels in Chinese full/half marathons. By doing so, we aim to contribute to the improvement of running shoe design by taking into account gender-specific and other individual characteristic demands".

## Methods

### Study design and participants

This cross-sectional study was conducted at the Hangzhou Marathon held by the China Athletics Association (Hangzhou, China) in November 2019. The basic inclusion criteria were: above 18 years old, demonstrating regular participation in long-distance running by engaging in the activity at least four times per week for the past six months, and having participated in at least one competition of more than 5 km, including both full marathon (42.195 km) and half marathon (21.0975 km) races. The exclusion criteria were: lower limb surgery or neurological injury.

### Sample size calculation

The sample size for this study was determined using the online Sample Size Calculator (Raosoft Inc., Seattle, WA, USA, raosoft.com). Considering a 5% margin of error, 95% confidence interval, and 50% response distribution, a sample size of 381 was recommended. It is worth noting that approximately 36,000 runners were enrolling in the marathon's competitions. A total of 822 runners were approached, and 626 runners returned their responses and consented to participate in the study, resulting in a response rate of 76.2%.

### Instruments and data collection

Data were collected through a supervised questionnaire that consisted of four sections. The questionnaire was categorized into four sections: (1) participant profile, (2) importance of shoe properties, (3) functional evaluation of shoe properties, and (4) importance ranking of shoe properties. All questionnaires were conducted after the participants finished the competition.

In the first section, participant profiles were obtained, including information such as gender, age, body height, body weight, race distance (full Marathon (42.195 km) or Half Marathon (21.0975 km)), and finish time.

The second section assessed the importance of various shoe properties as common requirements during running. The evaluated variables included forefoot curvature, forefoot bending stiffness, forefoot elasticity, heel curvature, heel cup, heel height, heel cushioning, midfoot anti-twist, midsole hardness, midsole thickness, outsole grip, guidance line, insole shape, upper breathability, upper elasticity, carbon fiber plate, shoelace, and shoe mass. Participants indicated their preferences on a 5-point Likert scale, ranging from 1 (Strongly unimportant) to 5 (Extremely important).

In the third section, participants were asked to evaluate whether specific shoe properties improve running performance or prevent sports injuries. The shoe properties assessed were the same as those in section two, and participants provided ratings using references A (Not important for running performance or preventing injuries), B (Important for running performance), C (Important for prevention of injuries), and D (Important for both running performance and prevention of injuries). The fourth section involved participants ranking the importance of shoe properties, and they selected the top three properties they deemed most important.

This study referred to the “Chinese Athletics Association Marathon Runners Level Evaluation Standards,” and the participants were classified into the following age groups: 18–29 years, 30–34 years, 35–39 years, 40–44 years, 45–49 years, 50–54 years, 55–59 years, 60–64 years, and 65 + years. Furthermore, each participant’s finish time was divided into the following performance groups: elite-level (87 runners), first-level (191 runners), second-level (210 runners), and third-level (138 runners) (As shown in Supplementary Table S1).

### Ethical considerations

The research protocol was reviewed and approved by the Li Ning Institutional Ethics Committee in accordance with the principles of the Declaration of Helsinki (approval code: LN-IRB-2019-003). Prior to participation, all participants were provided with detailed information regarding the purpose and content of the study. Informed consent was obtained from each participant. The research did not involve human clinical trials or animal testing.

### Data validity and collection

To ensure the consistency and reliability of the factor loadings, Cronbach's α coefficient was employed in this study, resulting in a value of 0.874, which indicated acceptable reliability of the questionnaire. The suitability of the data for factor analysis was assessed using the Bartlett spherical test and the Kaiser–Meyer–Olkin (KMO) test. The KMO value of 0.905 indicated that the questionnaire data were suitable for factor analysis. Furthermore, the Bartlett's test result (X^2^ = 3017.032, *df* = 153, *P* = 0.000) confirmed the necessity of the analysis.

The questionnaire was administered in the field, and participants completed it under the supervision of researchers who provided guidance to ensure the validity of the data. Researchers explained the definitions of footwear and foot-related terminology to avoid misunderstandings, particularly for participants with limited knowledge of footwear construction. Additionally, researchers ensured that participants did not provide random or missing answers, thus maintaining the questionnaire's quality. All questionnaires were completed after participants finished the competition.

### Data analysis

Descriptive statistics were used to describe the characteristics of the participants in the first section of the study. The Kolmogorov–Smirnov test was conducted on the data from the second and third sections, which revealed that the data did not conform to a normal distribution (*P* < 0.05). Therefore, non-parametric tests were used for further analysis. The Mann–Whitney U test was employed to analyze gender differences in the “Importance of shoe properties” section, and Fisher's Exact Test of Cross tabulation and Chi-square test were used for the analysis of the “Functional evaluation of shoe properties” section. Two-way ANOVA was used to analyze the interaction characteristics of gender and race within the context of our cross-sectional survey investigating gender differences in footwear characteristics between half and full marathons in China. The significance level was set at *P* < 0.05. All statistical analyses were performed using SPSS 21.0 (SPSS Inc., Chicago, IL, USA). All figures in this study were created using Origin 2021 (OriginLab Corporation, Northampton, MA, USA).

## Result

### Characteristics of the participants

A total of 626 questionnaires were collected in this study. The basic information of the participants is presented in Table 1, and all respondents gave informed consent and participated voluntarily. As shown below, most runners were males (76.2%), male and female participants in the full marathon were older than the half marathon, and females had lower body mass index (BMI) values than males.

**Table 1:** Characteristics of participants.

Gender	Male (n = 478)		Female (n = 148)	
Race	Half marathon (n = 129)	Full marathon (n = 349)	Half marathon (n = 79)	Full marathon (n = 69)
	Mean ± SD	Mean ± SD	Mean ± SD	Mean ± SD
Age (yr.)	35.4 ± 8.3	37.4 ± 9.6	37.3 ± 9.1	41.3 ± 9.9
Body height (cm)	173.3 ± 5.5	172.1 ± 5.5	160.1 ± 5.0	161.1 ± 5.3
Body weight (kg)	70.8 ± 11.7	66.6 ± 9.0	54.7 ± 8.8	54.0 ± 11.3
BMI (kg/m^2^)	23.6 ± 3.6	22.3 ± 3.3	21.1 ± 3.0	21.0 ± 4.4

Furthermore, this study used two-way ANOVA to analyze the interaction characteristics of gender and race in this survey, and found that there was no interaction between gender and race items on BMI [F_(1,622)_ = 1.789, *P* = 0.182, η^2^ = 0.002]. The main effect analysis showed that gender (F_(1,622)_ = 34.290, *P* < 0.001, η^2^ = 0.052) and race events [F_(1,622)_ = 1.789, *P* < 0.05, η^2^ = 0.008)] had significant effects on BMI, respectively. For race items, the BMI values of males in both the full marathon and half marathon were significantly higher than that of females (*P* = 0.001, 0.000). For gender, males who participated in the half marathon had a significantly higher BMI value than the full marathon (*P* = 0.001), but there was no significant difference in females.

### Importance of shoe properties

In Table 2, females were more concerned about upper elasticity than males, and females’ demand for running shoes was generally higher than males. The Mann–Whitney U test found no gender differences in evaluating the importance of shoe properties by elite-level runners in the full marathon. Compared with first-level male runners, females rated forefoot bending stiffness and upper elasticity as higher importance (*P* = 0.044, 0.001). For second-level runners, females reported higher importance of midsole hardness and upper elasticity than males (*P* = 0.024, 0.007). In addition, the importance scores of upper elasticity and shoelace in the third-level female runners were significantly higher than those of the male runners (*P* = 0.043, 0.046).

**Table 2:** Gender differences in full-marathon participants’ perceptions of the importance of shoe properties (Mean ± SD).

Shoe function	Elite-level		*P*	First-level		*P*	Second-level		*p*	Third-level		*P*
	Male	Female		Male	Female		Male	Female		Male	Female	
Forefoot curvature	3.83 ± 0.72	3.33 ± 1.49	0.549	3.54 ± 0.81	3.80 ± 0.63	0.104	3.46 ± 0.84	3.35 ± 0.70	0.509	3.32 ± 0.87	3.73 ± 0.77	0.103
Forefoot bending stiffness	3.97 ± 0.81	3.17 ± 1.07	0.054	3.74 ± 0.95	4.16 ± 0.61	0.044*	3.82 ± 0.89	4.00 ± 0.72	0.370	3.60 ± 0.82	3.87 ± 0.62	0.258
Forefoot elasticity	4.23 ± 0.78	3.50 ± 1.26	0.129	4.00 ± 0.92	4.16 ± 0.78	0.482	3.95 ± 0.80	4.04 ± 0.75	0.658	3.77 ± 0.82	3.73 ± 0.77	0.624
Heel curvature	3.69 ± 0.93	3.00 ± 1.00	0.170	3.37 ± 0.90	3.56 ± 0.80	0.341	3.50 ± 0.86	3.43 ± 0.71	0.572	3.43 ± 0.88	3.33 ± 0.94	0.876
Heel cup	3.60 ± 0.82	3.50 ± 1.26	0.782	3.82 ± 0.82	3.60 ± 0.75	0.204	3.88 ± 0.82	3.65 ± 0.81	0.219	3.43 ± 0.88	3.60 ± 1.02	0.675
Heel height	3.80 ± 0.86	4.17 ± 0.37	0.301	3.68 ± 0.96	3.60 ± 0.75	0.631	3.60 ± 0.86	3.78 ± 1.02	0.259	3.50 ± 0.92	3.87 ± 0.72	0.167
Heel cushioning	4.33 ± 0.89	4.17 ± 1.07	0.814	4.34 ± 0.78	4.48 ± 0.64	0.486	4.40 ± 0.73	4.39 ± 0.64	0.795	4.37 ± 0.58	4.20 ± 0.91	0.773
Midfoot anti-twist	4.00 ± 0.83	3.50 ± 1.26	0.394	3.93 ± 0.87	4.12 ± 0.86	0.242	4.08 ± 0.87	4.17 ± 0.76	0.714	3.90 ± 0.85	3.67 ± 0.94	0.317
Midsole hardness	4.01 ± 0.78	4.17 ± 0.69	0.700	3.91 ± 0.82	4.24 ± 0.81	0.052	3.89 ± 0.90	4.35 ± 0.70	0.024	3.78 ± 0.84	3.87 ± 0.88	0.700
Midsole thickness	3.86 ± 0.80	3.33 ± 1.25	0.327	3.69 ± 0.83	4.04 ± 0.77	0.053	3.60 ± 0.85	3.96 ± 0.75	0.072	3.70 ± 0.84	3.67 ± 0.87	0.771
Outsole grip	4.29 ± 0.76	3.50 ± 1.26	0.102	4.21 ± 0.81	4.36 ± 0.62	0.503	4.14 ± 0.77	4.48 ± 0.50	0.072	3.93 ± 0.93	4.07 ± 0.85	0.744
Guidance Line	3.67 ± 0.86	3.00 ± 1.29	0.190	3.66 ± 0.86	3.92 ± 0.84	0.234	3.70 ± 0.82	4.04 ± 0.81	0.082	3.45 ± 0.90	3.20 ± 0.65	0.170
Insole shape	3.84 ± 0.92	3.17 ± 0.69	0.078	3.65 ± 0.81	3.68 ± 0.88	0.902	3.54 ± 0.86	3.83 ± 0.82	0.130	3.28 ± 0.82	3.47 ± 0.62	0.477
Upper breathability	4.10 ± 0.86	3.83 ± 0.90	0.516	4.11 ± 0.73	4.04 ± 0.66	0.514	4.16 ± 0.72	4.04 ± 0.55	0.325	3.93 ± 0.70	4.00 ± 0.73	0.787
Upper elasticity	4.03 ± 0.93	3.33 ± 1.11	0.127	3.74 ± 0.91	4.36 ± 0.56	0.001*	3.63 ± 0.92	4.17 ± 0.56	0.007*	3.53 ± 0.87	4.07 ± 0.77	0.043*
Carbon fiber plate	4.19 ± 0.87	3.67 ± 0.94	0.195	3.84 ± 0.97	3.84 ± 0.92	0.845	3.92 ± 0.85	3.74 ± 0.94	0.450	3.55 ± 0.94	3.73 ± 0.77	0.597
Shoelace	3.71 ± 0.93	3.67 ± 0.47	0.790	3.61 ± 0.94	3.68 ± 1.12	0.383	3.47 ± 0.77	3.65 ± 0.91	0.248	3.38 ± 0.80	3.93 ± 0.85	0.046*
Shoe mass	4.46 ± 0.73	4.33 ± 0.75	0.662	4.39 ± 0.77	4.44 ± 0.50	0.863	4.33 ± 0.67	4.57 ± 0.50	0.159	4.32 ± 0.65	4.33 ± 0.70	0.866

*Indicates a significant difference, *P* < 0.05*.*

For half-marathon runners, there were no gender differences in the evaluation of shoe properties’ importance between elite-level and second-level runners, and the differences were mainly found in first- and third-level participants. Table 3 showed that the importance score of forefoot elasticity for first-level female runners was significantly lower than that of males (*P* = 0.034). Third-level female runners rated upper elasticity as more important than males (*P* = 0.017), while third-level males reported higher importance of carbon fiber plate and shoe mass (*P* = 0.028, 0.022).

**Table 3:** Gender differences in half-marathon participants’ perceptions of the importance of shoe properties (Mean ± SD).

Shoe function	Elite-level		*P*	First-level		*P*	Second-level		*p*	Third-level		*P*
	Male	Female		Male	Female		Male	Female		Male	Female	
Forefoot curvature	3.63 ± 0.86	3.33 ± 0.47	0.510	3.54 ± 0.78	3.54 ± 0.76	0.960	3.40 ± 0.79	3.47 ± 0.62	0.714	3.54 ± 0.80	3.59 ± 0.78	0.826
Forefoot bending stiffness	3.38 ± 0.48	4.00 ± 0.00	0.077	3.93 ± 0.75	3.75 ± 0.60	0.198	3.75 ± 0.90	3.77 ± 0.96	0.859	3.85 ± 0.84	3.82 ± 0.89	0.963
Forefoot elasticity	3.75 ± 0.43	4.00 ± 0.82	0.623	4.18 ± 0.66	3.79 ± 0.71	0.034*	3.87 ± 0.94	3.87 ± 0.99	0.923	4.12 ± 0.67	3.82 ± 1.03	0.400
Heel curvature	3.38 ± 0.70	4.00 ± 0.82	0.323	3.36 ± 0.77	3.42 ± 0.70	0.951	3.46 ± 0.84	3.17 ± 0.69	0.137	3.29 ± 0.89	3.45 ± 0.72	0.560
Heel cup	3.75 ± 0.66	3.67 ± 0.47	0.909	3.29 ± 0.96	3.79 ± 0.82	0.072	3.48 ± 1.05	3.70 ± 0.74	0.555	3.78 ± 0.84	3.45 ± 0.84	0.153
Heel height	3.50 ± 0.87	3.67 ± 0.47	0.742	3.46 ± 0.91	3.67 ± 0.80	0.616	3.44 ± 0.93	3.60 ± 0.76	0.617	3.68 ± 0.75	3.55 ± 0.72	0.367
Heel cushioning	4.50 ± 0.71	4.33 ± 0.47	0.567	4.25 ± 0.69	4.25 ± 0.60	0.850	4.27 ± 0.96	4.37 ± 0.71	0.962	4.44 ± 0.66	3.95 ± 1.19	0.145
Midfoot anti-twist	4.38 ± 0.86	4.33 ± 0.47	0.735	3.68 ± 0.93	3.92 ± 0.64	0.435	3.73 ± 1.04	3.70 ± 0.69	0.551	4.05 ± 0.82	3.86 ± 1.01	0.562
Midsole hardness	3.75 ± 1.09	4.33 ± 0.47	0.456	3.75 ± 0.83	3.96 ± 1.02	0.271	3.90 ± 0.74	3.90 ± 0.75	0.846	3.93 ± 0.64	3.86 ± 1.01	0.864
Midsole thickness	3.25 ± 0.83	3.33 ± 0.47	0.722	3.43 ± 1.02	3.75 ± 0.83	0.357	3.50 ± 0.84	3.63 ± 0.60	0.571	3.49 ± 0.74	3.77 ± 0.79	0.173
Outsole grip	3.88 ± 1.05	3.33 ± 0.47	0.394	4.21 ± 0.56	3.88 ± 0.73	0.087	4.00 ± 0.90	3.83 ± 0.90	0.380	4.24 ± 0.73	3.86 ± 1.18	0.340
Guidance Line	3.88 ± 0.78	3.67 ± 0.47	0.741	3.57 ± 0.78	3.54 ± 0.71	0.863	3.42 ± 0.84	3.40 ± 0.92	0.933	3.63 ± 0.69	3.64 ± 0.71	0.974
Insole shape	3.75 ± 0.97	4.00 ± 0.00	0.737	3.25 ± 0.87	3.54 ± 0.64	0.328	3.37 ± 1.06	3.43 ± 0.80	0.980	3.59 ± 0.91	3.59 ± 0.78	0.847
Upper breathability	3.88 ± 0.33	4.00 ± 0.00	0.540	3.82 ± 0.71	3.92 ± 0.70	0.898	3.87 ± 1.02	3.93 ± 0.89	0.903	4.02 ± 0.78	4.00 ± 1.09	0.582
Upper elasticity	4.13 ± 0.78	4.33 ± 0.47	0.741	3.82 ± 0.80	3.83 ± 0.69	0.774	3.58 ± 0.93	3.90 ± 0.79	0.123	3.61 ± 0.85	4.09 ± 0.90	0.017*
Carbon fiber plate	3.50 ± 1.22	4.00 ± 0.00	0.515	3.75 ± 0.87	3.71 ± 0.61	0.797	3.62 ± 0.98	3.40 ± 0.71	0.291	3.73 ± 0.77	3.32 ± 0.55	0.028*
Shoelace	3.50 ± 1.41	3.67 ± 0.47	0.981	3.61 ± 0.90	3.58 ± 0.81	0.814	3.35 ± 0.96	3.23 ± 0.76	0.609	3.49 ± 0.83	3.36 ± 0.93	0.659
Shoe mass	3.88 ± 1.27	4.67 ± 0.47	0.323	4.43 ± 0.49	4.21 ± 0.58	0.186	3.98 ± 0.99	4.00 ± 0.77	0.718	4.37 ± 0.79	3.82 ± 1.07	0.022*

*Indicates a significant difference, *P* < 0.05.

### Functional evaluation of shoe properties

The Fisher’s Exact Test was used to compare males’ and females’ functional evaluation of shoe properties.

**Figure 1:**
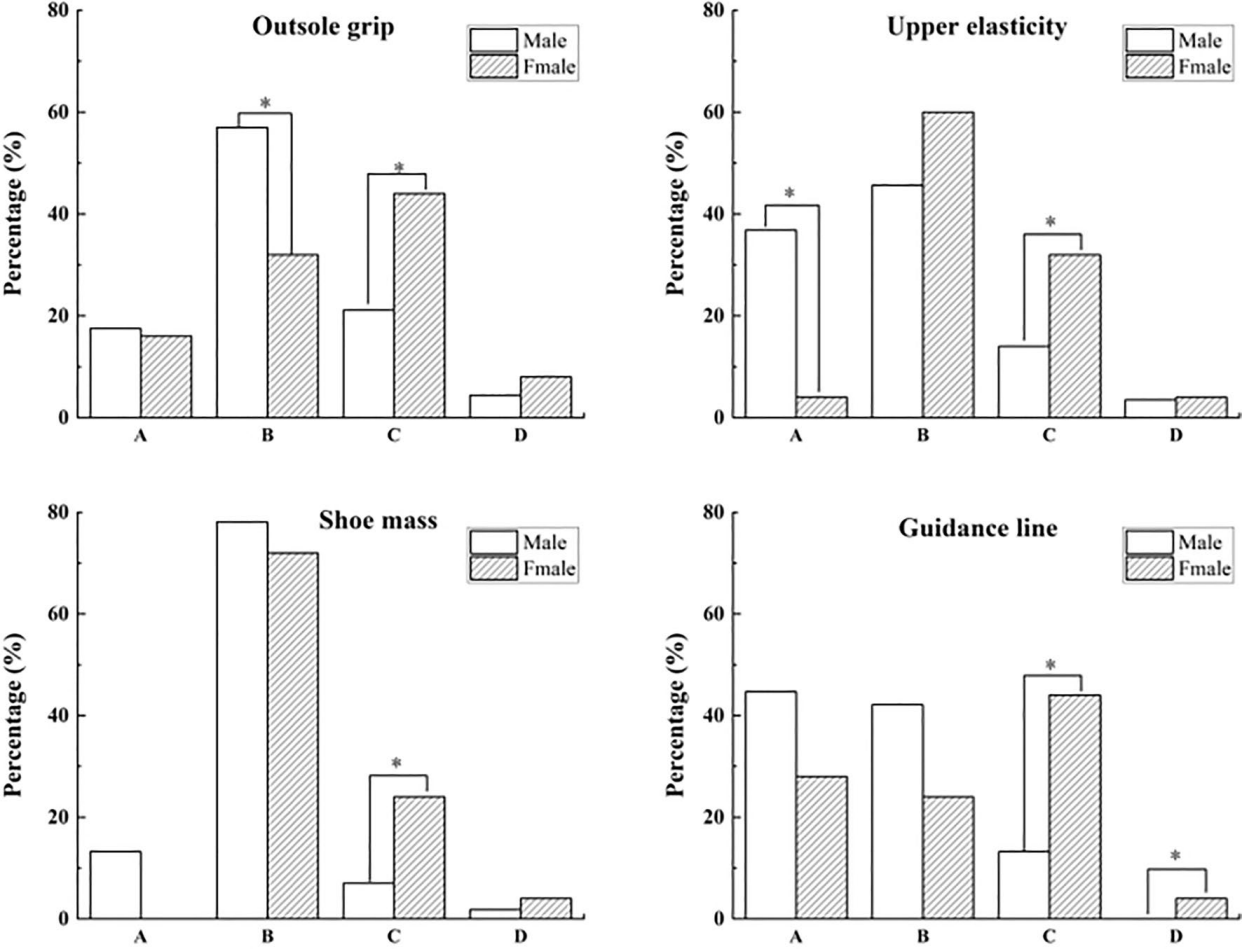
Gender differences in shoe properties functional perception of first-level participants in the full marathon. *Note*: (**A**) Not important for running performance and prevent injuries, (**B**) Important for running performance, (**C**) Important for prevent injuries, (**D**) Important for both running performance and prevent injuries. *Indicates a significant difference, *P* < 0.05.

This study found no gender differences in elite-level runners’ functional evaluations of shoe properties for full-marathon runners. There were significant gender differences in functional assessments of first-level runners in outsole grip, upper elasticity, shoe mass, and guidance line (*P* = 0.048, 0.002, 0.015, 0.001), as shown in Fig. 1.

Further conducted pairwise comparisons found that for outsole grip, 57% of males believed that this property was important in improving running performance, significantly higher than 32% of females (Fig. 1). Conversely, 44% of females thought the outsole grip was important for preventing sports injuries, more than 21.1% of males. Compared with 4% of females, 35% of males believed that the upper elasticity was neither beneficial for improving running performance nor preventing sports injuries. However, 32% of women felt the upper elasticity was important for preventing sports injuries, significantly higher than 14% of males. In addition, 7% of males thought shoe mass was important for preventing sports injuries, markedly less than 24% of females. 44% of females believe that the importance of the guidance line was reflected in preventing sports injuries, and 4% of females considered that this property could prevent sports injuries and improve running performance, which was significantly higher than that of males.

**Figure 2:**
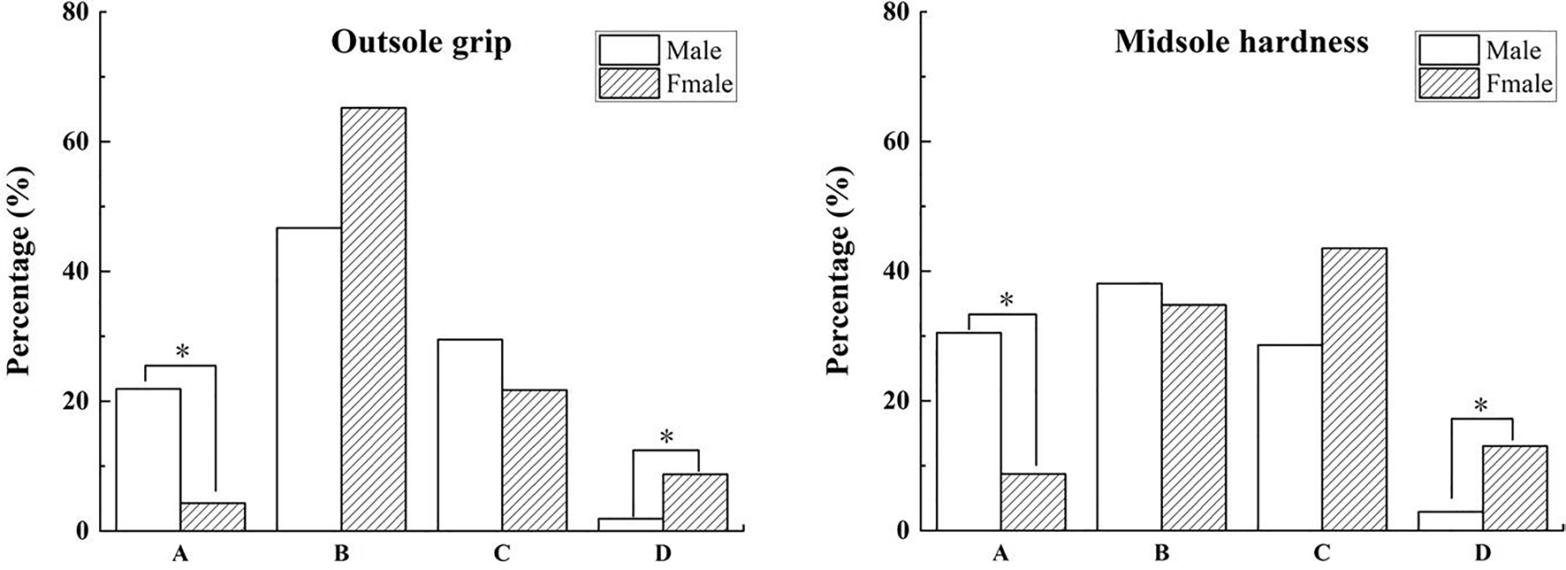
Gender differences in functional perception of shoe properties second-level participants in the full marathon. *Note*: (**A**) Not important for running performance and prevent injuries, (**B**) Important for running performance, (**C**) Important for prevent injuries, (**D**) Important for both running performance and prevent injuries. *Indicates a significant difference, *P* < 0.05.

Second-level participants’ functional evaluation of shoe properties found significant gender differences in outsole grip and midsole hardness (*P* = 0.046, 0.025), as shown in Fig. 2. For the outsole grip, 21.9% of males reported that the property was not crucial for running performance and injury prevention, and only 4.3% of females agreed with this, a significant difference. In contrast, 8.7% of females rated the characteristic as necessary for running performance and injury prevention, significantly more than 1.9% of males. Their evaluation of the function of midsole hardness was similar, males (30.5%) who rated that midsole hardness was not crucial for both running performance and injuries prevention significantly over females (8.7%), and females who considered that midsole hardness was necessary for both running performance and injuries prevention (13%) were significantly more than males (2.9%).

Fisher’s Exact Test showed no gender differences in functional evaluations of shoe characteristics between elite and second-level runners for half-marathon participants. However, gender differences existed between first-level and third-level runners.

**Figure 3:**
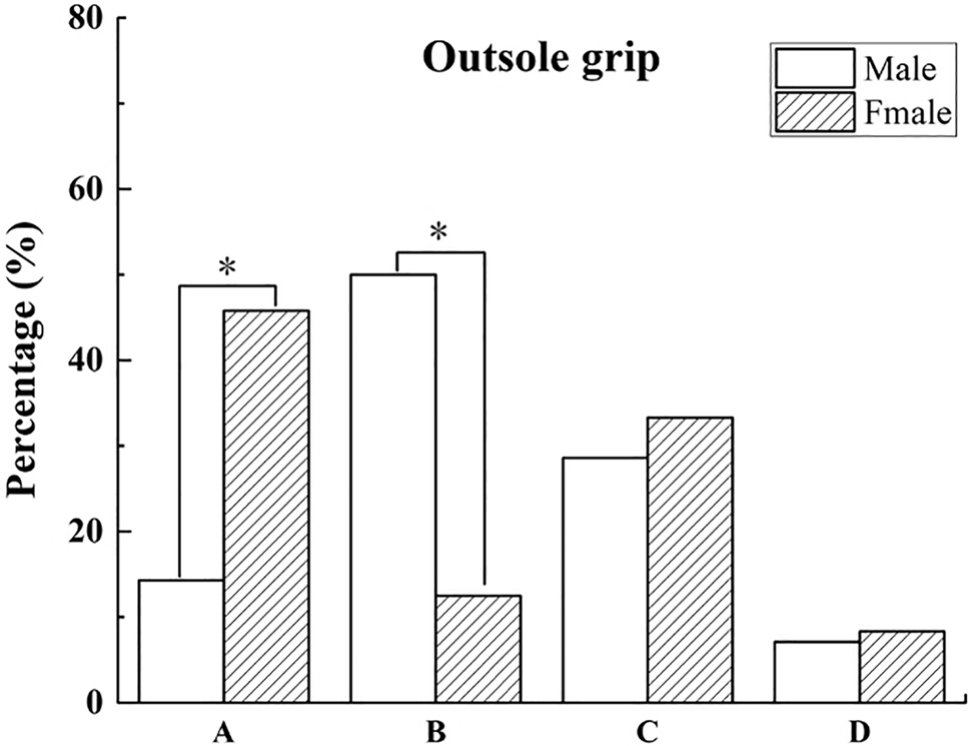
Gender differences in functional perception of shoe properties first-level participants in the half marathon. *Note*: (**A**) Not important for running performance and preventing injuries, (**B**) Important for running performance, (**C**) Important for preventing injuries, (**D**) Important for both running performance and preventing injuries. *Indicates a significant difference, *P* < 0.05.

Specifically, there was a significant gender difference (*P* = 0.012) in the functional evaluation of outsole grip for first-level runners in the half marathon. A pairwise comparison found that 45.8% of females and 14.3% of males rated this feature unimportant for running performance and injury prevention (Fig. 3). The proportion of females was significantly higher than that of males. In addition, 50% of males considered that the property of outsole grip was essential to running performance, significantly more than 12.5% of women, which was statistically significant, as shown in Fig. 3.

In addition, there was a significant gender difference in functional evaluations of upper elasticity and forefoot bending stiffness among third-level runners (*P* = 0.011, 0.002). 46.3% of males and 13.6% of females thought upper elasticity was unrelated to running performance or injury prevention. However, 18.2% of females rated upper elasticity as necessary for injury prevention, significantly more than 2.4% of males.

**Figure 4:**
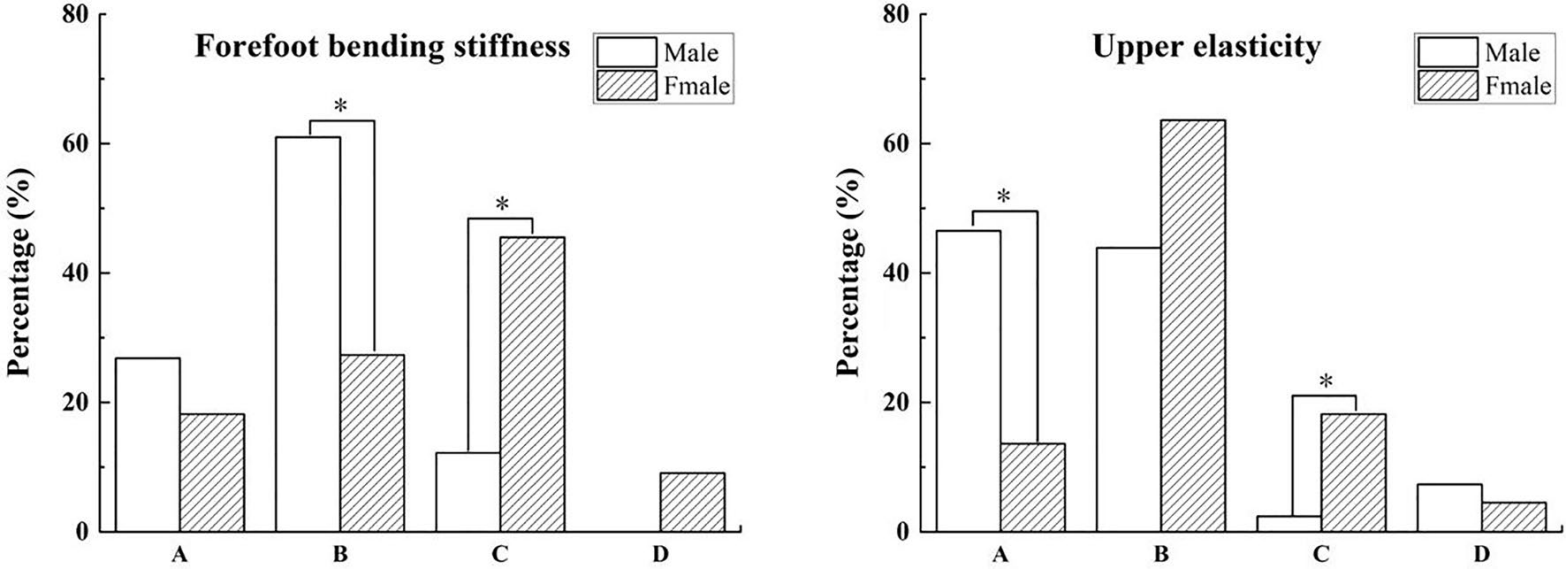
Gender differences in functional perception of shoe properties of third-level participants in the half marathon. *Note*: (**A**) Not important for running performance and preventing injuries, (**B**) Important for running performance, (**C**) Important for preventing injuries, (**D**) Important for both running performance and preventing injuries. *Indicates a significant difference, *P* < 0.05.

Furthermore, 61% of males and 17.3% of females considered forefoot bending stiffness unimportant for running performance and injury prevention, indicating a statistically significant difference. However, 12.2% of males reported this function as important for injury prevention, significantly less than 45.5% of females. Compared to 0% of males, 9.1% of females reported that this feature was important for running performance and injury prevention, indicating a significant difference, as shown in Fig. 4.

### Importance ranking of shoe properties

This study used descriptive statistics to conduct frequency statistics on the importance of shoe characteristics ranked by males and females in the full marathon and half marathon, respectively. Both males and females agreed that “heel cushioning” was the most critical running shoe feature, but there were differences in the ranking of other shoe features.

**Figure 5:**
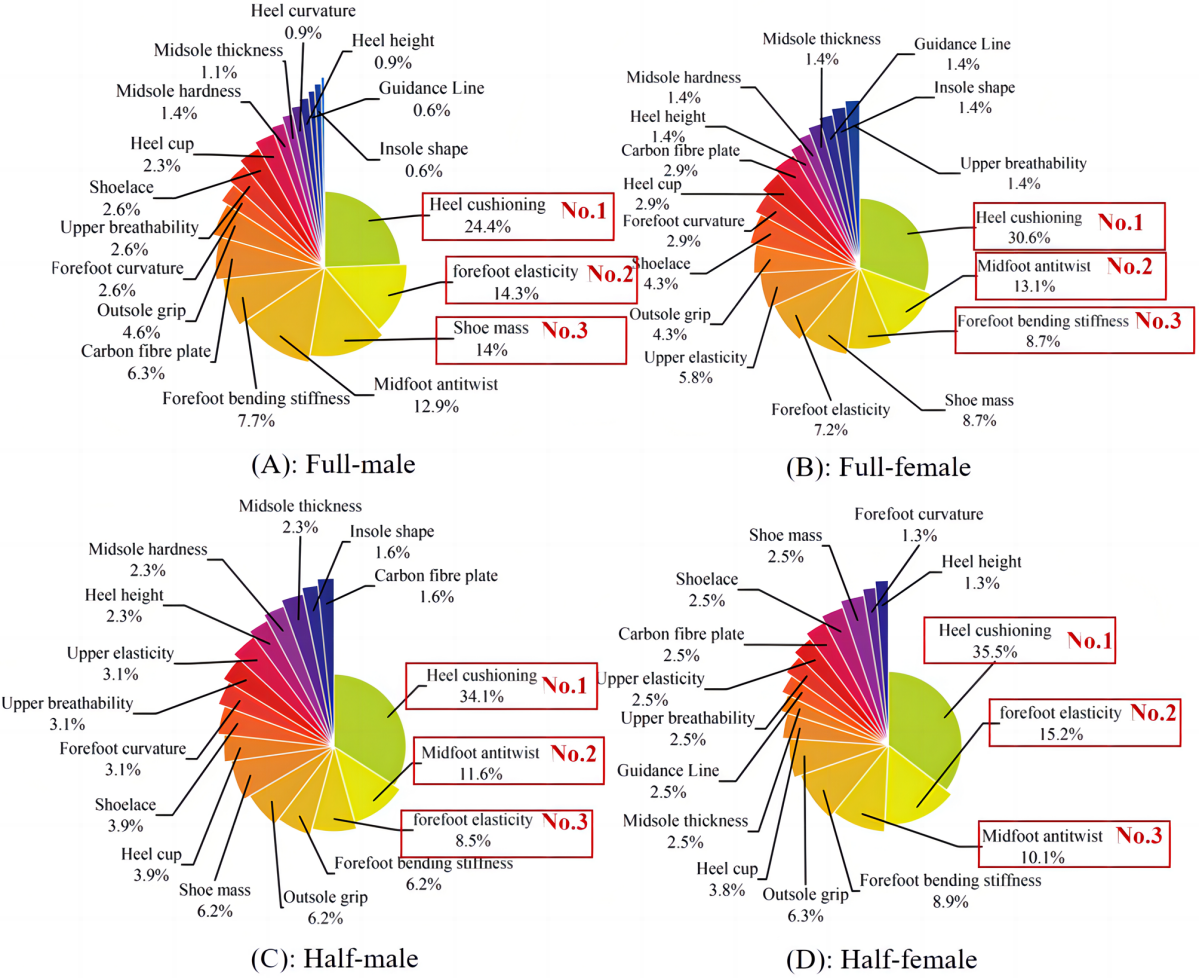
Ranking of the importance of shoe properties. (**A**)- Full-male; (**B**)- Full-female; (**C**)- Half-male; (**D**)- Half-male;. The red box represents the top three ranked shoe characteristics.

Specifically, the three properties that male full marathon participants rated as the most important were “heel cushioning,” “forefoot elasticity,” and “shoe mass.” The top three shoe traits for females were “heel cushioning,” “midfoot anti-twist,” and “forefoot bending stiffness,” as shown in Fig. 5.

In addition, half-marathon participants identified "heel cushioning," "midfoot anti-twist," and "forefoot elasticity" as the three most important characteristics of shoes, as shown in Fig. 5. Furthermore, upon analyzing the data separately for male and female participants, we found that female participants rated "shoe mass" as one of their top three preferred characteristics, while male participants favored "forefoot elasticity" and "forefoot bending stiffness" as their preferred features".

## Discussion

In this study, we observed a significantly higher number of male participants completing marathon races compared to females. According to the "2019 China Marathon Big Data Report" released by the Chinese Athletics Association, the number of participants increased by 14.28% in 2019 compared to 2018. Among them, the number of male participants in China was considerably higher than females. However, in the half marathon races, the number of female participants exceeded that of males, aligning with the findings of our study but contrasting with the trend observed in the United States^28^. These findings highlight the gender disparities in marathon participation in China, with a higher proportion of males in the full marathon category and a higher engagement level of females in the half marathon category.

To better understand the reasons behind these gender differences, it is important to consider factors such as motivation and demographics. The "2019 China Marathon Big Data Report" revealed that male full marathon runners in China were more motivated, accounting for 74.63% of all male participants. In our study, we found a similar trend, with 73% of male participants completing the full marathon. In contrast, the percentage of male participants in the half marathon was 27%, while females accounted for 53% of all female participants. These findings suggest that in Chinese marathon events, there is a significantly higher number of male participants in the full marathon category compared to females, while female participants demonstrate a higher level of engagement in the half marathon category.

Furthermore, our study explored the age distribution of marathon participants and found that female participants were older than male participants, with an average age of over 35 years old. This result is consistent with the analysis of the age group of Chinese marathon runners from 2016 to 2019, indicating that the primary finishers of Chinese marathons are predominantly middle-aged individuals. Several factors, including physical and mental needs, social influence, and disposable time, may contribute to this age distribution^29^.

In addition to age, we also examined the influence of gender and age on athletic performance. It was observed that regardless of gender, participants who completed the full marathon were older compared to those who completed the half marathon. This finding suggests that older participants are more inclined to participate in longer endurance sports, reflecting their greater emotional control and sense of responsibility for completing tasks^5,30^.

Another aspect we investigated was the relationship between participants' BMI and their involvement in marathon races. We found that the BMI values of male full marathon participants were significantly lower than those of half marathon participants, and the BMI values of female participants were significantly lower than those of male participants. Previous cross-sectional studies have suggested that BMI contributes to the risk of running-related injuries in population samples^31–33^. More specifically, a low BMI even increases female runners’ risk of lower extremity injury^31^. Specifically, a low BMI increases the risk of lower extremity injury in female runners due to their tendency to have lower body fat percentages compared to non-marathon females^32,33^. Although some studies have shown no direct association between participants' BMI and injury risk, considering BMI as a potentially modifiable risk factor becomes relevant if it is influenced by marathon activity^34^.

Moving on to the preferences for shoe characteristics among elite runners, we found no gender differences in these preferences in both the half and full marathon categories. This observation indicates that elite runners, regardless of gender, possess a comprehensive understanding of shoes after extensive training sessions and consistently prioritize shoe properties that enhance athletic performance^35,36^. Their knowledge enables them to select more suitable running shoes that align with their specific running requirements^35,36^.

Forefoot bending stiffness is a crucial factor in footwear performance development^37^, and it plays a significant role in maintaining both comfort and performance in running shoes^38^. Furthermore, it has been observed that increasing the forefoot bending stiffness in footwear can reduce the extent of metatarsophalangeal joint extension during movement^39^. In our study, female marathon participants consistently ranked forefoot bending stiffness as their third most important consideration, indicating a higher expectation for this characteristic compared to males. These findings highlight the significance of forefoot bending stiffness in meeting the specific needs and preferences of female runners. Previous studies on gender differences in Chinese foot shape show that Chinese females have a lower first-toe height than males^19^. Therefore, females wearing running shoes with the same forefoot bending stiffness at the same running interface need to generate a larger metatarsophalangeal joint moment, which is more likely to increase the risk of injury of metatarsal stress fractures^39^. The subjective reports of female runners also underscore this point.

Additionally, female full marathon runners expressed a higher level of concern about the upper elasticity of running shoes compared to males. Previous studies have shown that upper elasticity is a critical factor affecting comfort and may impact shoe choice preferences^40^. Biomechanical studies have also shown that changes in the upper elasticity can even lead to changes in running patterns^41^. This preference for footwear comfort aligns with the notion that for runners, emotional value and overall experience hold significance, alongside athletic performance^42^.

Moreover, our study analyzed the preferences of runners based on their finishing times and identified specific characteristics that different levels of runners prioritize. For instance, female three-level finishers, who took the longest to finish the race, emphasized the necessity of shoelaces. This preference aligns with the idea that shoelaces allow for a more comfortable shoe fit, enabling runners to adjust the tightness to obtain a custom fit that accommodates the shape of their foot^43^. Therefore, the fit design of shoelaces is vital for marathon runners, as increased long-distance running time may lead to increased foot movement in the shoe, and ill-fitting laces can cause blisters and subungual hematomas^44,45^.

In this study, full-marathon first-level males emphasized forefoot elasticity significantly more than females^46^. Studies have shown that changing the flexibility of the forefoot area of a running shoe can provide a greater range of motion in the forefoot and increase activation of the calf muscles^47,48^. Chen et al.’s research showed that increasing the forefoot elasticity of the soles of running shoes can reduce the activity of muscles^49^, thereby reducing energy consumption and improving exercise performance. In the half marathon, the first-level male participants also emphasized forefoot elasticity compared with females, which was consistent with the statistics for full-marathon participants.

A study has examined the impact of shoe mass on preference, performance, and biomechanical variables^50^. In another study, it was found that for every 100g reduction in shoe weight, running economy improved by 1% and running performance improved by 0.7%^51^. In this research, third-level males reported higher importance of shoe mass. Specifically, heavier footwear reduced comfort in second and third-level runners and increased energy requirements at all running levels, potentially reducing preference^52^. Heavier shoes had a significant effect on ankle angle, ankle moment^53^ and plantar pressure (second and third-level runners)^54^, which is consistent with the results of this study.

In the “Functional evaluation of shoe properties” part, females were more concerned about whether these properties were necessary for injury prevention, while males were more concerned about the importance of shoe properties to running performance, which may be because females’ shoe lasts usually downsized versions of males’ shoe lasts, and women rarely buy suitable shoes when purchasing running shoes, and inappropriate shoes will increase the risk of injury during running^16^. However, males can usually buy shoes that fit their feet and preference, which can improve sports performance.

Heel cushioning was reported in this study as the most critical function for all participants, which is an essential function of running shoes. Robbins et al. suggest that the increased cushioning in running shoes can attenuate the perceived magnitude of forces acting on the foot plantar surface^55^. The study by Mark et al. showed that runners (rearfoot strike pattern) used the same pair of running shoes to run 480 km, and the amount of heel cushioning of the rear running shoes would be reduced by 16% to 33%^56^. Based on previous research results by Taunton et al., heel support and cushioning function will decrease with running shoes, and the risk of long-distance running injury will increase^57^. Therefore, stabilizing the heel cushioning performance of running shoes is significant for preventing injuries. In addition, male and female participants in the same schedule have different attributes of shoes ranked second and third, and the same-gender participants of different programs also have different opinions. Based on our findings and previous studies, it is important to consider specific characteristic designs in running shoes for different genders and different race distances. For example, our results had shown that female runners may benefit from shoe designs that address factors such as heel cushioning, midfoot anti-twist, and shoe mass. On the other hand, male runners in marathon races have shown a preference for shoe characteristics such as heel cushioning, forefoot elasticity, and forefoot bending stiffness. These examples highlight the need for gender-specific and race-specific considerations in running shoe design.

## Limitations

Our study has several limitations that should be acknowledged when interpreting the findings and considering their generalizability. Firstly, it is important to note that participants in our study did not wear the same shoes, which may have resulted in variations in wearing experiences and shoe preferences^58^. This heterogeneity in footwear selection could introduce bias and potentially influence participants' perceptions of shoe properties, thereby affecting the validity of our findings. Therefore, caution should be exercised when generalizing the results to populations where participants wear standardized shoes.

Secondly, our study recruited a relatively smaller number of elite players, which limits the generalizability of the findings to the elite athlete population^59,60^. Elite athletes often possess unique characteristics and preferences that differ from recreational runners, and their perceptions of shoe properties may vary significantly. Hence, the applicability of our results to elite-level marathon runners should be interpreted with caution.

Additionally, we acknowledge that the COVID-19 pandemic has had a significant impact on various aspects of society, including the field of sports and athletics. Unfortunately, our study did not assess data from the years 2020–2022, which coincided with the height of the pandemic. This represents a limitation in capturing the potential influence of the pandemic on Chinese marathon runners and their perceptions.

## Conclusion

There were no gender differences between elite players’ demand for running shoes, but significant gender differences were found between genders at other running levels. Both males and females agreed that “heel cushioning” was the most critical running shoe feature. Females pay more attention to the protection brought by shoes, while males pay attention to the sports performance of shoes.

In conclusion, our study underscores the importance of considering gender and distance factors when designing running shoes. The distinct characteristics demanded by male and female runners, along with the variations related to different running distances, emphasize the need for customization and optimization in the development of running footwear. We believe that our findings contribute valuable knowledge to the field and have practical implications for the running shoe industry.

### Supplementary Information

Supplementary Table S1.

## Data Availability

All data generated or analysed during this study are included in this published article.
