# Supplementary material for: Gender differences in footwear characteristics between half and full marathons in China: a cross-sectional survey
Source: Sci Rep. 2023 Aug 10;13:13020. doi: 10.1038/s41598-023-39718-x (PMC10415251; doi:10.1038/s41598-023-39718-x)
Supplement: Supplementary file 1 — Supplementary Table S1. [file 41598_2023_39718_MOESM1_ESM.pdf]

Supplementary Table S1 Chinese Athletics Association Marathon Runners Level Evaluation Standards (Time).

**Levels**	**Race**	**Gender**	**Age groups (years)**								
			**18- 29**	**30- 34**	**35- 39**	**40- 44**	**45- 49**	**50- 54**	**55- 59**	**60- 64**	**65+**
Elite	Half	Female	1h 49m	1h 50m	1h 51m	1h 52m	1h 53m	1h 54m	1h 55m	1h 56m	2h 03m
		male	1h 34m	1h 35m	1h 36m	1h 37m	1h 38m	1h 39m	1h 40m	1h 41m	1h 48m
	Full	Female	3h 48m	3h 49m	3h 50m	3h 51m	3h 52m	3h 53m	3h 54m	4h 04m	4h 30m
		male	3h 24m	3h 25m	3h 26m	3h 27m	3h 28m	3h 29m	3h 33m	3h 39m	3h 50m
Level 1	Half	Female	2h 11m	2h 12m	2h 13m	2h 14m	2h 15m	2h 16m	2h 17m	2h 18m	2h 19m
		male	1h 51m	1h 52m	1h 53m	1h 54m	1h 55m	1h 56m	1h 57m	1h 58m	2h 05m
	Full	Female	4h 25m	4h 26m	4h 27m	4h 28m	4h 29m	4h 32m	4h 34m	4h 41m	4h 30m
		male	4h 03m	4h 04m	4h 05m	4h 06m	4h 07m	4h 08m	4h 09m	4h 14m	3h 50m
Level 2	Half	Female	2h 31m	2h 32m	2h 33m	2h 34m	2h 35m	2h 36m	2h 37m	2h 38m	2h 41m
		male	2h 14m	2h 15m	2h 16m	2h 17m	2h 18m	2h 19m	2h 17m	2h 21m	2h 29m
	Full	Female	5h 17m	5h 18m	5h 19m	5h 20m	5h 21m	5h 22m	5h 23m	5h 26m	5h 27m
		male	4h 53m	4h 54m	4h 55m	4h 56m	4h 57m	4h 58m	4h 59m	5h 04m	5h 16m
Level 3	Half	Female	3h	3h	3h	3h	3h	3h	3h	3h	3h
		male	3h	3h	3h	3h	3h	3h	3h	3h	3h
	Full	Female	6h	6h	6h	6h	6h	6h	6h	6h	6h
		male	6h	6h	6h	6h	6h	6h	6h	6h	6h

Note: The runner classification criteria are based on participants completing the race within or equal to the time indicated in the table. More information can be found at: https://www.athletics.org.cn/bulletin/hygd/mls/2023/0323/464400.html
